# Integrated Approach to Extract and Purify Proteins from Honey by Ionic Liquid-Based Three-Phase Partitioning

**DOI:** 10.1021/acssuschemeng.2c01782

**Published:** 2022-06-24

**Authors:** Matheus M. Pereira, Sónia N. Pedro, Maria V. Quental, Aminou Mohamadou, João A. P. Coutinho, Mara G. Freire

**Affiliations:** † CICECO − Aveiro Institute of Materials, Chemistry Department, University of Aveiro, 3810-193 Aveiro, Portugal; ‡ Institut de Chimie Moléculaire de Reims (ICMR), CNRS UMR 7312, UFR des Sciences Exactes et Naturelles, Université de Reims Champagne-Ardenne, 51100 Reims, France

**Keywords:** Ionic liquid, three-phase partitioning, major royal jelly proteins, antioxidants, extraction, purification

## Abstract

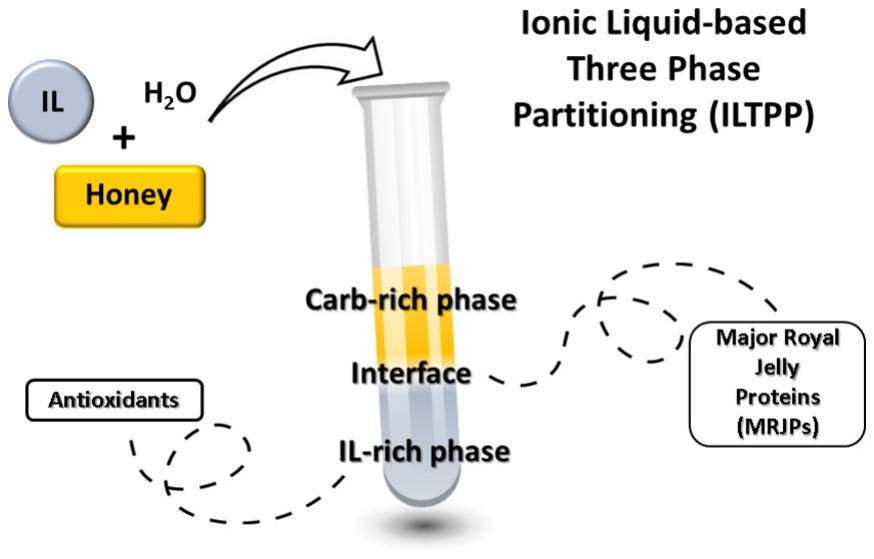

The purification of value-added compounds by three-phase partitioning (TPP) is a promising alternative to conventional processes since the target compound can be easily recovered from the liquid–liquid interphase. Although this technique has been successfully applied to the recovery of proteins, the minimization of the use of salts and solvents must be pursued to improve the overall process sustainability. Accordingly, we have here investigated the use of biobased glycine-betaine ionic liquids (IL) directly with honey, a carbohydrate-rich matrix, as phase-forming components of TPP systems. These ILTPP systems were applied in the purification of major royal jelly proteins (MRJPs) from honey. The results obtained show that MRJPs mostly precipitate in the ILTPP interphase, with a recovery yield ranging between 82.8% and 97.3%. In particular, MRJP1 can be obtained with a purity level up to 90.1%. Furthermore, these systems allow the simultaneous separation of antioxidants and carbohydrates to different liquid phases. The proposed approach allows the separation of proteins, antioxidants, and carbohydrates from honey in a single step, while using only ILs and a real carbohydrate-rich matrix, thus being sustainable TPP processes.

## Abstract 2

The proposed approach allows the separation of proteins, antioxidants, and carbohydrates from honey in a single step, while using only ionic liquids and a real carbohydrate-rich matrix, thus being sustainable TPP processes.

## Introduction

The increasing global concern about health and the environment has intensified measures to guarantee that food, pharmaceuticals, and cosmetics’ commercialization is safely conducted.^1−3^ These procedures intend to maximize public health advantages and environmental benefits provided by these products. Therefore, the use of value-added compounds from natural sources and the avoidance of adulterated products are in high demand.^4,5^ The extraction and purification of these natural compounds, such as proteins, have been achieved from complex matrix sources like milk^6^ and soybean^7^ or even meeting the biorefinery concept of food waste^8^ and microalgae.^9^ Additionally, these proteins can present important biological activities, being used as markers in quality control and to verify the product authenticity, as happens for honey.^10,11^ Honey is a natural supersaturated solution of sugars produced by honeybees.^12^ In addition to its high carbohydrate content, it is also rich in valuable phenolic compounds, aliphatic acids, vitamins, amino acids, and inorganic compounds.^13^ Honey proteins, particularly, display interesting properties such as anti-inflammatory,^14^ antimicrobial,^15^ and anticancer^16^ activities. Several proteins with molecular weights ranging from 22 to 75 kDa are present in honey, in concentrations ranging from 0.1% to 0.5%.^17^ Among these are major royal jelly proteins (MRJP), like the MRJP1 (accounting for 48% of water-soluble RJ proteins) which is likely to promote liver regeneration and to have a cytoprotective action on hepatocytes.^18^ MRJP3 can exhibit potent immunoregulatory effects *in vitro* and *in vivo*,^19^ and both MRJP4 and MRJP5, less abundant proteins, are important sources of essential amino acids.^20^ Despite their applications in food, nutraceutical, and cosmetic contexts with extensive health benefits, their use is still limited by their extraction and purification processes, which remain challenging.

Several studies on the precipitation of proteins from honey have been reported by the addition of ammonium sulfate, sodium tungstate, or trichloroacetic acid.^21^ Dialysis, centrifugation, and chromatographic techniques (e.g., adsorption, ion exchange, and affinity chromatography) have also been attempted for honey proteins separation from sugars and other small metabolites.^11^ More recently, these proteins were extracted using saturated solutions of phenol.^22^ In general, multistep approaches are required, often involving the use of compounds that are either toxic or may compromise the proteins integrity. Due to the limited number of methods applied for honey proteins extraction and recovery, alternative options must be considered.

Given the advantages of the three-phase partitioning (TPP) technique, this can be a promising option to attain the described goal. TPP is a method to isolate proteins that takes benefit from the ability of some aqueous two-phase system (ATPS) phase-forming components to induce the precipitation of target proteins on an interphase.^23^ Several proteins have been isolated from complex matrixes using TPP.^24,25^ Despite the efficiencies of previously reported TPP-based systems, most studies focused on the ability of TPP to separate target products using organic solvents, mainly t-butanol.^26^ To move further on this field, as well as to extend the applicability of these systems, it is essential to develop of novel TPPs for the separation and purification of proteins from real matrices using more benign and sustainable phase-forming components. In this sense, ionic liquid-based TPPs (ILTPPs) were recently proposed to promote the precipitation of target proteins between the two phases of IL-based ATPS.^23^ These systems maintain the IL-based ATPS advantages and may improve the product recovery. ILTPPs have been used in the separation of monoclonal antibodies,^27^ whey protein,^28^ and nonsteroidal anti-inflammatory drugs (NSAIDs).^29^

Furthermore, the uses of ILs for the extraction of antibiotics, such as sulfathiazole and chloramphenicol from honey samples have been previously reported, confirming the compatibility of ILs with this matrix.^30,31^

The search for more benign ILs has led to the synthesis of ILs derived from natural sources, resulting in solvents with a more biocompatible character. Among these biobased ILs, the use of glycine–betaine-derived ILs (AGB-ILs) has enabled the possibility to develop even more sustainable and safe processes.^27,32^ However, none of these works explored the ability of combining these ILs with real matrices used as phase-forming components. Herein, we propose a novel ILTPP approach that uses a carbohydrate-rich matrix, honey, and a biobased IL to achieve liquid–liquid demixing and simultaneous protein precipitation. This ILTPP can be applied in the selective extraction and purification of not only proteins from honey but also other relevant compounds such as antioxidants and carbohydrates. This integrated approach can be envisioned to be applied in the fractionation of these compounds for nutraceutical and cosmetic applications. Ultimately, we confirm the sustainability of the process showing the possibility to recover and reuse ILs in the system.

## Material and Methods

### Materials

The commercial ILs used in this study, namely, 1-butyl-3-methylimidazolium bromide ([C_4_mim]Br, 99 wt %) and 1-butyl-3-methylimidazolium trifluoromethanesulfonate ([C_4_mim][CF_3_SO_3_], 99 wt %), were purchased from Iolitec (Heilbronn, Germany). Tetrabutylammonium chloride ([N_4444_]Cl, ≥ 97 wt %), tetrabutylammonium bromide ([N_4444_]Br, 98 wt %), and tetrapropylammonium bromide ([N_3333_]Br, 98 wt %) were purchased from Sigma-Aldrich (St. Louis, MO, USA). Triisobutyl(methyl)phosphonium tosylate ([P_*i*(444)1_][Tos], >98 wt %), tetrabutylphosphonium chloride ([P_4444_]Cl, >98 wt %), tetrabutylphosphonium bromide ([P_4444_]Br, >96 wt %), and tributylmethylphosphonium methylsulfate ([P_4441_][MeSO_4_], >99 wt %), were kindly provided by Cytec Ind (NJ, USA).

**Figure 1:**
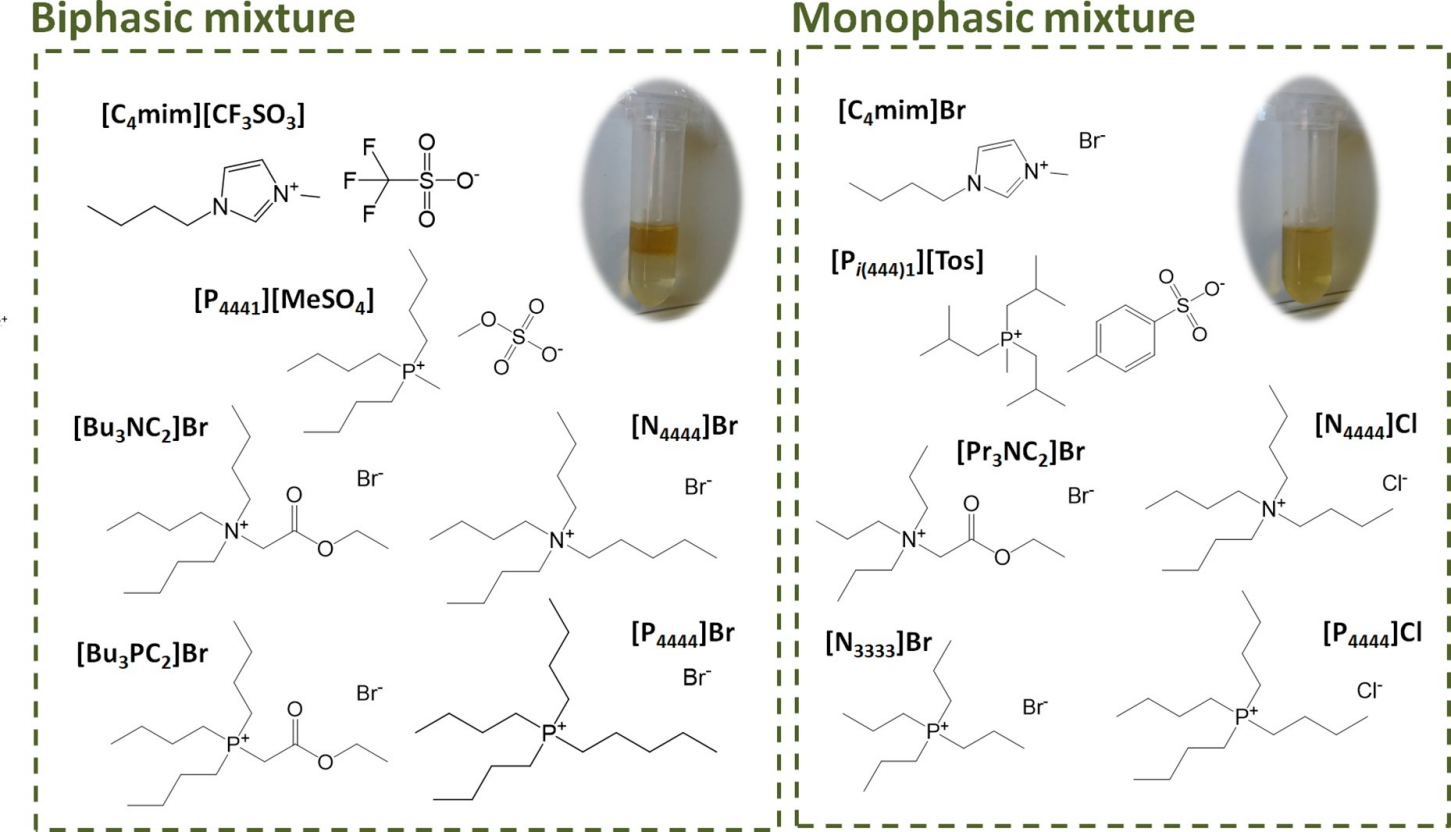
Chemical structures of the studied ILs and their ability for liquid–liquid demixing in a ILTPP composed of 25 wt % IL, 15 wt % water, and 60 wt % honey.

The glycine–betaine analogue ILs (AGB-ILs) were synthesized by us according to previously reported protocols.^33^ The AGB-ILs tri(*n*-propyl)[2-ethoxy-2-oxoethyl]ammonium bromide ([Pr_3_NC_2_]Br), tri(*n*-butyl)[2-ethoxy-2-oxoethyl]ammonium bromide ([Bu_3_NC_2_]Br), and tri(*n*-butyl)[2-ethoxy-2-oxoethyl]phosphonium bromide ([Bu_3_PC_2_]Br) were synthesized by the reaction of ethyl 2-bromoacetate and tri(*n*-propyl)amine, tri(*n*-butyl)amine, and tri(*n*-butyl)phosphine, respectively. All AGB-ILs were dried under vacuum for at least 72 h at 45 °C. After this procedure, the purity of each IL was checked by ^1^H and ^13^C nuclear magnetic resonance (NMR), being >98%. The NMR data of each AGB-IL are given in the Supporting Information. All ILs synthesized are solid at room temperature, yet water soluble. The materials used in the ILs synthesis were purchased from Sigma-Aldrich. The molecular structures of the ILs investigated are depicted in Figure 1. Commercial honey was purchased at a local market, presenting 17.2 wt % of water, 82.0 wt % of carbohydrates, and 0.4 wt % of proteins. The average antioxidant capacity of the honey used was 42.6 ± 2.6% for 20% w/v of honey aqueous solutions.

### Purification and Recovery of Proteins from Honey

The ILTPP mixture compositions applied in the recovery and purification of proteins from honey were chosen based on phase diagrams previously reported.^34^ An initial screening was performed to determine the best mixture composition to be used. ILTPP compositions ranging between 15 and 35 wt % IL and 50–70 wt % honey were studied. After selection of the best mixture, that composition was used to determine the best IL to develop the fractionation process of proteins from honey. Each mixture was vigorously stirred, centrifuged for 30 min (7500 rpm), and left to equilibrate for 10 min at 25 °C to allow complete phase separation and products partitioning/recovery. After, a careful separation of the phases was performed, and the amounts of total proteins in each phase were determined. Quantification was conducted using the Bradford’s method,^35^ by application of a calibration curve previously established with bovine serum albumin (BSA) (Figure S1, Supporting Information). UV–vis spectroscopy was carried out for quantification purposes, using a BioTek Synergy HT microplate reader at 595 nm (Biotek Instruments, Winooski, VT, USA). To eliminate the influence of the ILs and carbohydrates present on the protein concentration analysis, a blank control for each mixture was prepared and used. When preparing these mixtures, a solid interphase is created, mainly composed of proteins as discussed below. This phase was recovered, resuspended in a phosphate buffer solution (PBS), and the proteins contents quantified. At least three independent ILTPPs were prepared for each mixture and three samples of each phase quantified, allowing one to determine the associated uncertainty of the recovery yield and purity percentage of proteins.

The recovery yield of honey proteins, RY_PROT_%, was determined as the percentage ratio between the total amount of proteins in the interphase to that in the honey, according to eq 1
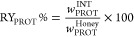
1where *w*_PROT_^INT^ and *w*_PROT_^Honey^ are the total weight of protein in the interphase and in honey, respectively.

Since the recovery yields were based on the total amount of protein extracted, the purity percentage of MRJ1 was determined according to sodium dodecyl sulfate polyacrylamide gel electrophoresis (SDS-PAGE) results, considering the intensity corresponding to the target protein and the total intensity corresponding to all proteins in the interphase (taking into account all the dilution factors applied to each solution), according to eq 2
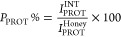
2where *I*_PROT_^INT^ and *I*_PROT_^Honey^ are the intensity corresponding to MRJ1 and the total intensity corresponding to all proteins present in the interphase, respectively.

In all systems, the top phase corresponds to the carbohydrate-rich phase, whereas the bottom phase is mainly composed of the IL and water.

### Sodium Dodecyl Sulfate Polyacrylamide Gel Electrophoresis (SDS-PAGE)

The protein profile of ILTPP interphase was investigated by SDS-polyacrylamide gel electrophoresis (SDS-PAGE) with Amersham ECL gel equipment (GE Healthcare Life Sciences, USA). ILTPP interphase was resuspended in a phosphate buffer solution and directly mixed with a Laemmli buffer (1:1, v/v) in the presence and absence of a reducing agent, dithiothreitol (DTT), and then heated at 90 °C for 5 min to complete denaturation and subjected to SDS-PAGE in 20% polyacrylamide gels. The proteins were stained with Coomassie Brilliant Blue G-250 for 2–3 h and then distained with a mixture of methanol, acetic acid, and distilled water (50:37:413, (v/v)) at room temperature. All gels were analyzed using the *ImageJ* analysis tool. The molecular weight marker used was the Amersham ECL rainbow full-range molecular weight marker (Merck, NJ, USA) with size ranges from 12 to 225 kDa.

### DPPH Radical-Scavenging Activity

The antioxidant content in each phase was determined using DPPH radical-scavenging activity. Each ILTPP phase was mixed with a methanolic solution containing DPPH radicals. The mixture was shaken vigorously and left to stand for 60 min in the dark (until stable absorption values were obtained). The reduction of the DPPH radical was determined by measuring the absorption at 517 nm. The radical-scavenging activity (RSA) for each phase (top and bottom phases) was calculated as a percentage of DPPH discoloration using eq 3
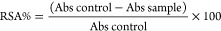
3where Abs control is the absorbance of the control, and Abs sample is the absorbance of the top and bottom phases. Aiming to eliminate the influence of the solvents (IL and carbohydrates), a blank control for each mixture was prepared and used. Gallic acid was used as the standard.

### Antioxidant Recovery

The recovery of the antioxidants present in the IL-rich phase was performed by solid-phase extraction using Oasis HLB cartridges previously washed with methanol (1 mL). Each IL-rich phase containing the honey antioxidants was passed through the column, to which the antioxidant compounds were adsorbed, followed by 1 mL of methanol. The antioxidant fraction was desorbed by addition of 1 mL of methanol. All fractions were collected and analyzed regarding the presence of IL and an antioxidant in each fraction. Antioxidants were quantified by the DPPH method and IL recovery, and absence in the antioxidant fraction was confirmed by high-performance liquid chromatography with diode array detection (HPLC-DAD) (Shimadzu, model PROMINENCE, Kyoto, Japan).

## Results and Discussion

### Purification and Recovery of Proteins from Honey

**Figure 2:**
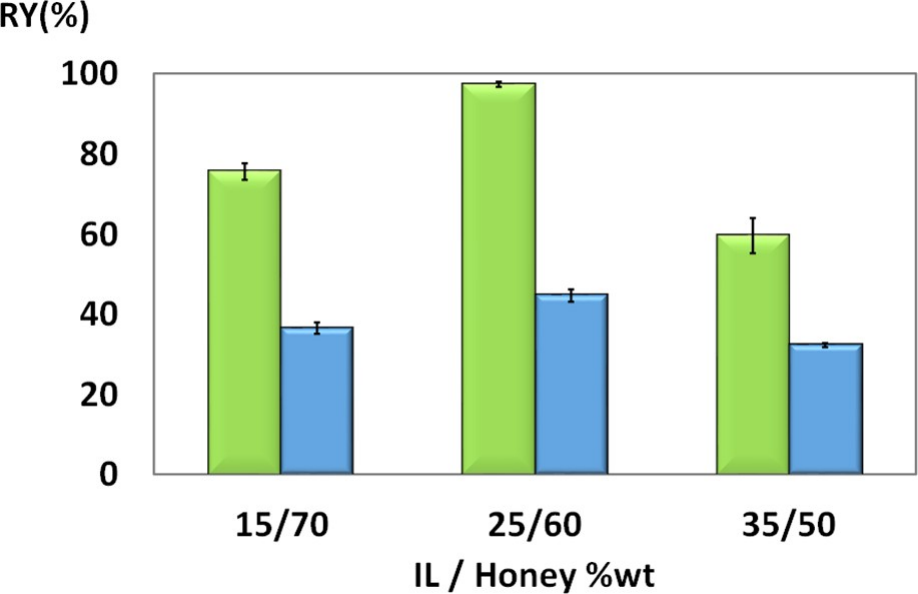
Recovery yield (RY%) of proteins (green bars) and antioxidants (blue bars) from honey, at different ILTPP compositions with [Bu_3_PC_2_]Br, at 25 °C: 15–35 wt % IL and 50–70 wt % honey.

To investigate the ability to extract and fractionate several honey compounds simultaneously we performed an initial screening. For this purpose, we fixed the IL studied, [Bu_3_PC_2_]Br, and tested ILTPP compositions ranging between 15 and 35 wt % IL and 50–70 wt % honey (Figure 2). This IL and these values were selected according to previous works regarding its ability to phase separate with aqueous solutions of carbohydrates.^34^

For all systems, a large amount of precipitated proteins at the interphase was observed. The protein quantification was carried out in top and bottom phases, and the precipitated proteins at the interphase were resuspended in a PBS for further analysis. The antioxidants were also quantified in both the top and bottom phases to evaluate which mixture had the higher ability to recover them. On the basis of the results presented in Figure 2, we have selected the mixture composed of 25 wt % IL, 60 wt % honey, and 15 wt % water, since it presented higher recovery yields for both proteins and antioxidants.

**Figure 3:**
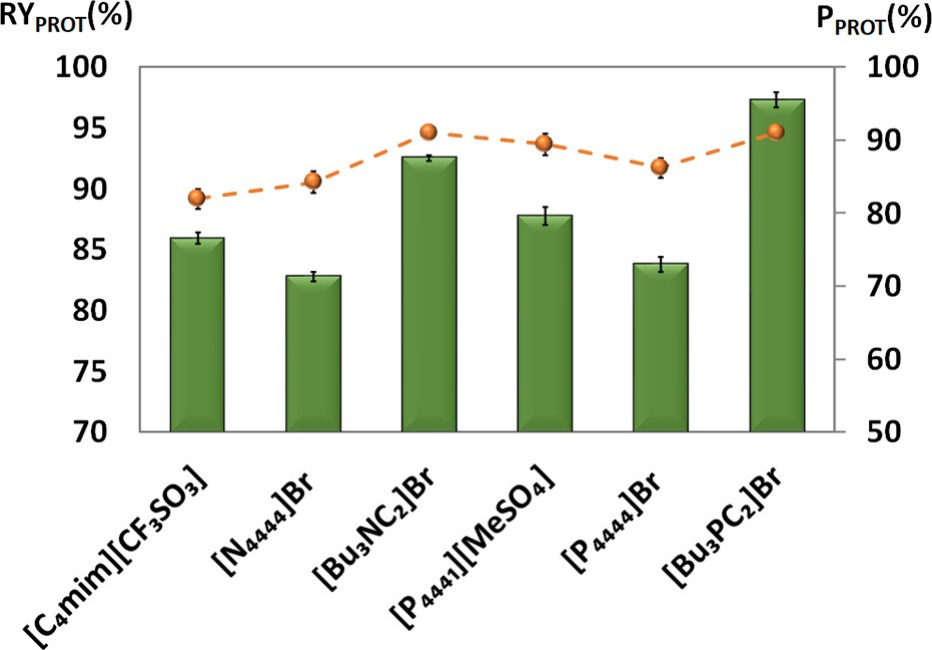
Recovery yield (RY_PROT_%, bars) and purification (*P*_PROT_, symbols) of proteins from honey in ILTPP composed of 25 wt % IL + 60 wt % honey +15 wt % water at 25 °C.

Following this, we start by evaluating the ability of several ILs to recover and purify proteins from honey. To this purpose, we studied ammonium- and phosphonium-based ILs, whose results are depicted in Figure 3. The recovery yield of total proteins at the interphase ranges between 82.8% and 97.3%. The ability to promote the protein recovery increased in the following order: [N_4444_]Br > [P_4444_]Br > [C_4_mim][CF_3_SO_3_] > [P_4441_][MeSO_4_] > [Bu_3_NC_2_]Br > [Bu_3_PC_2_]Br. Tetralkyl-based ILs are more hydrophobic than imidazolium.^36^ Due to this hydrophobicity, it is possible that specific interactions lead to an unfavorable environments for honey proteins, inducing their precipitation at the interphase. Considering the results here presented, these ILTPPs can act as an integrated extraction and purification processes in a single-step that can be easily scalable.

The extraction, purification, and isolation of honey proteins were previously investigated using physical and/or chemical methods.^37,38^ Ion exchange chromatograph and dialysis are usually required to obtain amylase from honey.^37^ Moreover, aiming to achieve honey protein isolation, a solution of sodium tungstate combined with sulfuric acid is required to induce the precipitation of honey proteins.^38^ However, this method requires not only the use of high temperatures but also sample centrifugation and washing until the supernatant becomes sugar free. The use of ILTPP for this purpose not only allows one to promote the precipitation of honey proteins between the two phases but also avoids the additional steps of removal of the initial matrix components, making it a one-step process.

**Figure 4:**
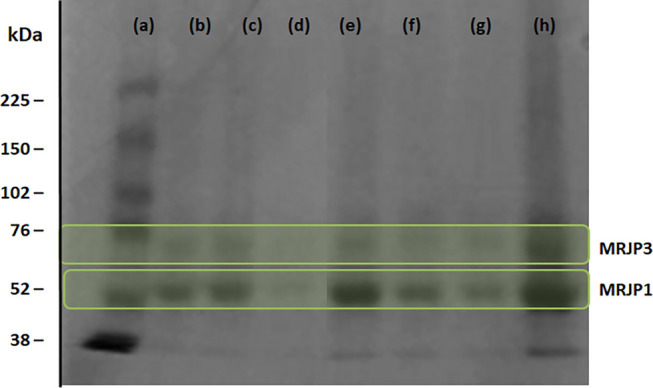
SDS-PAGE of the (a) protein molecular weight marker and precipitated proteins on the interphase and redissolved in a buffer solution of the ILTPP composed of (b) [C_4_mim] [CF_3_SO_3_], (c) [P_4444_]Br, (d) [P_4441_][MeSO_4_], (e) [Bu_3_PC_2_]Br, (f) [Bu_3_NC_2_]Br, (g) [N_4444_]Br, and (h) commercial honey.

Aiming to identifying the precipitate proteins, the ILTPP interphase was resuspended in a phosphate buffer solution and analyzed by SDS-PAGE. As shown in Figure 4, the proteins present in the studied honey, according to molecular weight markers, correspond to the major royal jelly proteins (MRJPs) MRJP1 and MRJP3. Likewise, according to band intensity, the system composed of 25 wt % [Bu_3_PC_2_]Br + 62.5 wt % honey, and 12.5 wt % water was the ILTPP that allowed the MRJP1 precipitation at the interphase with the highest purity level (90.1 ± 0.5%). The results show that ILTPP composed of ILs and honey can be applied for the extraction, purification, and isolation of MRJP1, with a recovery yield of 97.3% and high purity levels, despite the loss of the other less abundant proteins detected by UV–vis at the carbohydrate-rich phase.

### Recovery of Antioxidants from Honey

**Figure 5:**
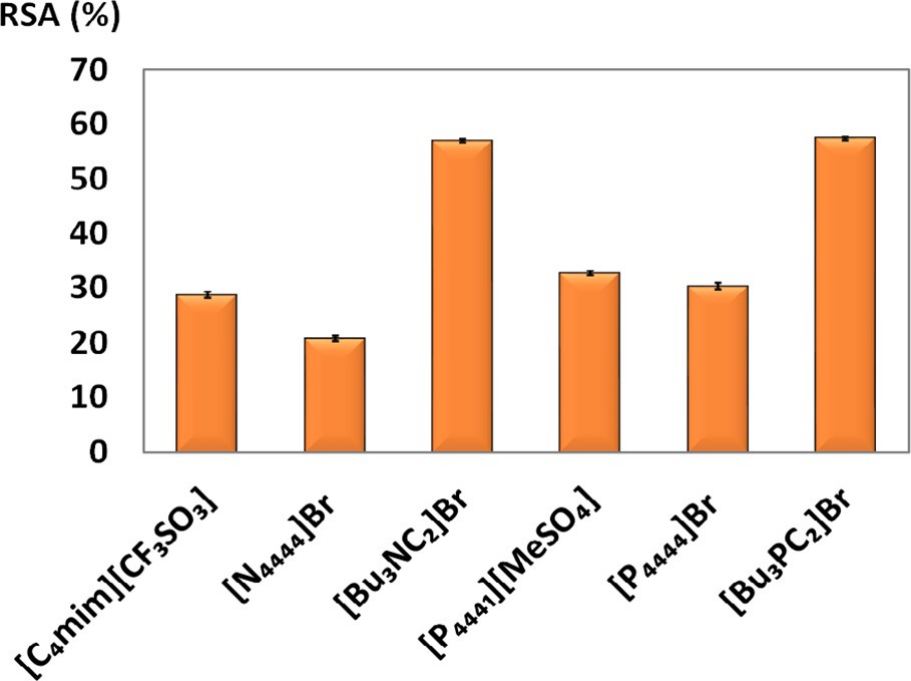
DPPH radical-scavenging activity (RSA%) of the IL-rich phase of ILTPP composed of 25 wt % IL + 60 wt % honey +15 wt % water at 25 °C.

To further explore the ability of the proposed ILTPP for the fractionation of valuable honey compounds, the contents of antioxidants were quantified in the phases by the DPPH radical-scavenging activity. The results obtained are displayed in Figure 5. In all systems using AGB-ILs ([Bu_3_PC_2_]Br and [Bu_3_NC_2_]Br), the DPPH radical-scavenging activity was higher in the IL-rich phase (the carbohydrate-rich phase for all systems studied presented negligible reactivity). On the other hand, for conventional ILs ([C_4_mim] [CF_3_SO_3_], [P_4444_]Br [P_4441_][MeSO_4_], and [N_4444_]Br), the antioxidants preferentially partition to the carbohydrate-rich phase.

The described results are in agreement with the literature. IL-based ATPS were previously employed for the extraction of antioxidants.^39,40^ Imidazolium-based ILs combined with inorganic salts were investigated in IL-based ATPS for the extraction of vanillin and gallic acid. Those works showed the partition of antioxidants to be controlled by the pH of the system.^40^ Other IL-based ATPS with citrate buffer were evaluated for the extraction of eugenol and propyl gallate, which also preferentially partitions to the IL-rich phase.^39^ The complete extraction of these antioxidants was obtained for cyclic ILs, whereas tetralkyl-based ILs presented lower extractions efficiencies.^39^

**Figure 6:**
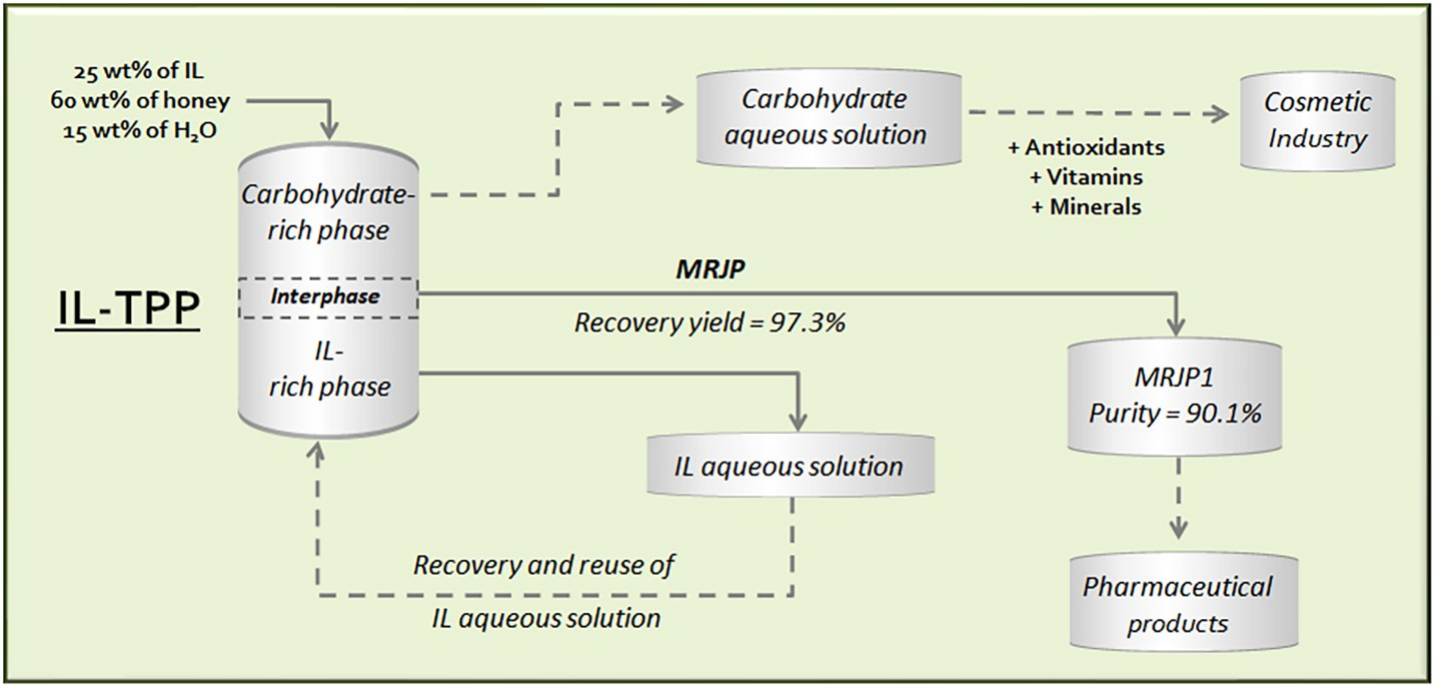
Flowchart of the integrated process for the extraction, purification, and isolation of valuable bioactive compounds from honey, including the IL recycling.

Aiming to present an integrated process that allows one to simultaneously recover and purify honey proteins, being able to selectively isolate carbohydrates and antioxidants in honey samples, we studied the use of ILTPP composed by ILs, as presented in Figure 6. To show the possibility to recycle and reuse the IL and to recover the antioxidants from the IL-rich phase, we carried out a solid-phase extraction, based on the process previously reported.^34^ We were able to recover with success the eluted IL and adsorb the antioxidant compounds onto the column, recovering them by elution. This process allowed us to recover up to 90% of the initial antioxidant content in the IL-rich phase (Table S2, Supporting Information). The possibility to successfully subject the IL-rich solution to an evaporation under vacuum at room temperature and its reuse in the system formation has been previously explored.^41^ Furthermore, this reuse without significant loss of extraction performance shows the feasibility of the recovery and reuse of the IL in the process. Since the protein in the interphase is in close contact with both the carbohydrate- and the IL-rich phases, minimal contaminations of both phases could occur, and to fully purify the protein, a simple additional step of dialysis can be envisaged.

The traditional MRJP recovery processes applied are in their majority based on hardazous volatile organic solvents.^37,38^ In addition, significant amounts of waste are produced due to the several steps required to achieve the extraction, purification, and isolation of target compounds, being overcome in this work by the one-step separation of proteins, antioxidants, and carbohydrates while using biobased ILs and a natural matrix. The ILTPP systems proposed here have the following advantages contributing to sustainability: (i) All phase-forming components used are from natural sources. (ii) A carbohydrate-rich matrix is used, which acts as a phase-forming component, requiring only the addition of IL and water. (iii) The ILTPP systems reported herein are capable of extracting, purifying, and isolating MRJP1, antioxidants, and carbohydrates from honey in one step. (iv) It can be a low-cost process due to the ability to reuse the applied ILs.^41^ In summary, the ILTPP systems here reported are a step forward in the improvement of traditional separation technologies.

## Conclusions

This work proposes an ionic liquid-based three-phase partitioning system composed of biocompatible ILs and honey, which contain carbohydrates acting as phase-forming components and simultaneously being the matrix where the target compounds are present, thus representing more sustainable TPP alternatives. Using the studied ILTPP systems, proteins from honey are precipitated at the interphase with high recovery yields (82.8%–97.3%) and purity (80.0%–90.1%) for MRJP1. The studied systems allow the simultaneous separation of proteins, antioxidants, and carbohydrates from honey in a single-step procedure. The ILTPP composed of 25 wt % of [Bu_3_PC_2_]Br + 62.5 wt % of honey and 12.5 wt % of water was the most efficient system, allowing the MRJP1 recovery at interphase with the highest purity level (90.1 ± 0.5%). Besides, the simultaneous separation of antioxidants and carbohydrates to different liquid phases was achieved, in a single step. The ILTPP systems here developed, being composed of biobased ILs and honey, are thus sustainable systems that could be used in the fractionation of valuable bioactive compounds from real matrices. Other carbohydrate-rich matrices should be investigated, such as milk or fermentation broths, in which only a phase-forming inducer needs to be added to create TPP and fractionate the target biocompunds.

## Supporting Information Available

Detailed information regarding AGB-ILs ^1^H and ^13^C nuclear magnetic resonance (NMR), recovery yield (RY_PROT_%) and purification (*P*_PROT_%) of MRJP and weight fraction compositions of ILTPP, DPPH radical-scavenging activity (RSA%) of IL-rich phase of ILTPP and antioxidant aqueous solution with ILs removed, and protein calibration curves determined by the Bradford’s method (PDF)
