# Supplementary material for: Integrated Approach to Extract and Purify Proteins from Honey by Ionic Liquid-Based Three-Phase Partitioning
Source: ACS Sustain Chem Eng. 2022 Jun 24;10(29):9275–81. doi: 10.1021/acssuschemeng.2c01782 (PMC9777929; doi:10.1021/acssuschemeng.2c01782)
Supplement: Supplementary file 1 — sc2c01782_si_001.pdf [file sc2c01782_si_001.pdf]

Integrated Approach to Extract and Purify Proteins from Honey by Ionic Liquid-Based Three-Phase Partitioning

Matheus M. Pereira[1], Sónia N. Pedro[1], Maria V. Quental[1], Aminou Mohamadou[2], João A. P. Coutinho[1] and Mara G. Freire[1*]

[1]CICECO-Aveiro Institute of Materials, Department of Chemistry, University of Aveiro, 3810-193 Aveiro, Portugal
[2]Université de Reims Champagne-Ardenne, Institut de Chimie Moléculaire de Reims (ICMR), CNRS UMR 7312, UFR des Sciences Exactes et Naturelles, Reims, France

*Corresponding author

Tel: +351-234401422; Fax: +351-234-370084; E-mail address: maragfreire@ua.pt

Contents:
Number of Pages: 5
Number of Figures: 1
Number of Tables: 2

Supporting Information

Section S1

Characterization of the synthetized AGB-ILs

Tri(n-propyl)[2-ethoxy-2-oxoethyl]ammonium bromide ([Pr_3NC_2]Br)

1H NMR (250 MHz, DMSO-d6): 0.7 (t, 9H), 1.1 (t, 3H), 1.55 (m, 6H), 4.00 (s, 2H) and 4.41 (4, 2H).

Tri(n-butyl)[2-ethoxy-2-oxoethyl]ammonium bromide ([Bu_3NC_2]Br)

1H NMR (250 MHz, DMSO-d6): 0.93 (t, 9 H), 1.25 (t, 3 H), 1.30 (q, 6 H), 1.62 (m, 6 H), 3.43 (m, 6 H), 4.23 (s, 2 H), and 4.38 (q, 2 H).

Tri(n-butyl)[2-ethoxy-2-oxoethyl]phosphonium bromide ([Bu_3PC_2]Br)

1H NMR (300 MHz, DMSO-d6): 0.93 (t, 9H), 1.25 (t, 3H), 1.30 (q, 6H), 1.62 (m, 6H), 3.43 (m, 6H), 4.23 (s, 2H), 4.38 (q, 2H).

Table S1

Recovery yield (*RY* $\%_{PROT}$) and purification (*P* $\%_{PROT}$) of MRJP and weight fraction compositions of the initial mixtures at 25 ºC.

IL	Weight fraction composition / (wt%)		$RY\ \%_{PROT} \pm \sigma$	$P\ \%_{PROT} \pm \sigma$
	IL	Honey		
$[C_4mim][CF_3SO_3]$	25.55 ± 0.02	60.61 ± 0.07	85.92 ± 0.47	81.96 ± 1.36
$[N_{4444}]Br$	24.36 ± 0.04	60.43 ± 0.03	82.78 ± 0.37	84.19 ± 1.48
$[Bu_3NC_2]Br$	25.37 ± 0.06	60.14 ± 0.09	92.53 ± 0.23	90.78 ± 1.49
$[P_{4441}][MeSO_4]$	25.05 ± 0.07	60.83 ± 0.09	87.75 ± 0.75	89.42 ± 1.48
$[P_{4444}]Br$	24.95 ± 0.02	60.86 ± 0.12	83.84 ± 0.60	80.02 ± 1.36
$[Bu_3PC_2]Br$	24.97 ± 0.04	60.96 ± 0.07	97.30 ± 0.61	90.98 ± 0.49

Table S2

DPPH radical-scavenging activity (RSA%) of IL-rich phase of ILTPP and antioxidant aqueous solution with IL removed.

	**IL-rich phase of ILTPP**	**Antioxidant aqueous solution with IL removed**
DPPH radical-scavenging activity (RSA%)	63.38 ± 0.52	55.55 ± 0.48

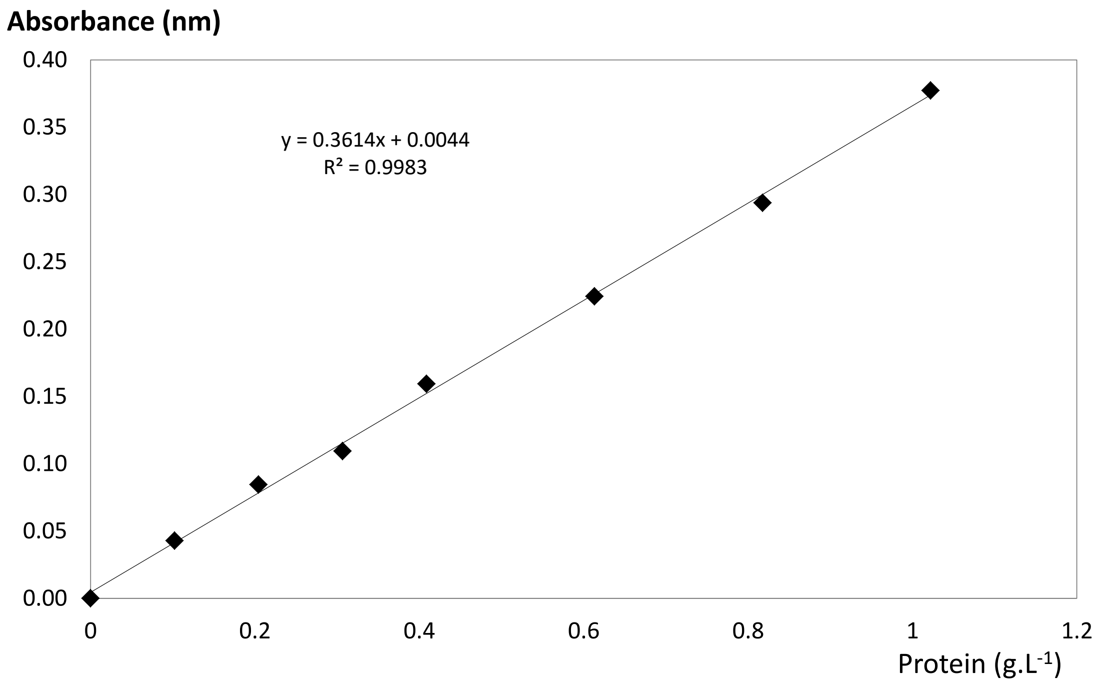

Figure S1. Proteins calibration curve (UV-Vis - 595 nm) determined by the Bradford's method.
